# A Scoping Review of Clinical Studies on Procedures of Ultrasound-Guided Injection to Ensure Hygiene and Safety

**DOI:** 10.3390/healthcare13101165

**Published:** 2025-05-16

**Authors:** Yujin Kweon, Goeun Jeong, Sungha Kim, Changsop Yang, Eunbyul Cho, Jungtae Leem

**Affiliations:** 1 College of Korean Medicine, Wonkwang University, Iksan 54538, Republic of Korea; heibrie00@gmail.com; 2 Easebody Korean Medical Clinic, Gwangju 61931, Republic of Korea; goeunjeong.kmd@gmail.com; 3 KM Science Research Division, Korea Institute of Oriental Medicine, Daejeon 34054, Republic of Korea; bozzol@kiom.re.kr (S.K.); yangunja@kiom.re.kr (C.Y.); 4 Department of Diagnostics, College of Korean Medicine, Wonkwang University, Iksan 54538, Republic of Korea; 5 Research Center of Traditional Korean Medicine, College of Korean Medicine, Wonkwang University, Iksan 54538, Republic of Korea; 6 Department of Il-won Integrated Medicine, Wonkwang University Korean Medicine Hospital, Iksan 54538, Republic of Korea

**Keywords:** ultrasound-guided injection, infection prevention, sterile technique, probe disinfection, procedural safety, scoping review

## Abstract

**Background**: Ultrasound guidance is widely used to enhance injection accuracy and safety. However, ultrasound-guided procedures require complex manipulation of both probe and needle. This simultaneous manipulation while maintaining sterility necessitates specific infection prevention protocols. This scoping review aimed to systematically investigate hygiene and safety procedures reported in clinical studies of ultrasound-guided injections. **Methods**: Following the Joanna Briggs Institute guideline, we conducted a systematic search of four databases (two English and two Korean) from inception to November 2023. Studies describing ultrasound-guided injection procedures with skin disinfection protocols were included. The extracted procedures were categorized and analyzed according to their timing (before, during, and after injection) and purpose. **Results**: Among 1728 studies identified, 86 met inclusion criteria. Notable variations were found in infection prevention practices, with only 5.81% reporting probe disinfection procedures and 27.91% documenting sterile probe cover use. Skin disinfection methods also varied, with iodophors (20.93%) and alcohol-based solutions (11.63%) being most common. Of studies describing ultrasound coupling agent procedures (26.74%), less than 20% specifically mentioned using sterile transmission agents. Documentation of temporal aspects of infection prevention was limited, with most studies not addressing precise timing of disinfection procedures or post-procedure probe reprocessing protocols. **Conclusions**: Our findings reveal considerable variation in infection prevention practices during ultrasound-guided injections and highlight gaps in documentation of hygiene protocols. These findings suggest the need for standardized, evidence-based protocols tailored to different anatomical sites and types of injections. Further research through expert consensus and real-world implementation is needed to develop and validate comprehensive guidelines for clinical practice.

## 1. Introduction

Ultrasound (US)-guided injections have increasingly gained popularity in clinical practice globally in recent years, owing to their ability to improve accuracy and safety by visualizing the needle tip and targeting anatomical structures in real time [1]. Systematic reviews have shown that US-guided injections are more accurate, indicating better efficacy than blind injections [2,3]. Particularly in South Korea, traditional landmark-based acupuncture techniques have rapidly transformed into direct imaging-guided procedures [4]. A recent survey revealed that the most common primary objective for using US guidance among Korean Medicine doctors (KMDs) was to “improve accuracy and efficacy of the procedure”. In particular, pharmacopuncture (the injection of herbal medicine extracts) accounted for approximately 80% of these US-guided interventions [5].

Despite the benefits of US-guided injections, the use of an US probe has added more safety concerns than previous blind injections. One major concern is the risk of infection. The US-guided procedure requires the practitioner to hold the US probe in one hand and the syringe needle in the other, while simultaneously coordinating the probe and needle. Needle and procedure site contamination can unintentionally occur due to the proximity of the probe and needle during the process. Furthermore, US scanning requires the use of transmission gel. However, the use of non-sterile gel on the probe may contaminate the disinfected procedure site while the probe is being moved for scanning [6,7]. In clinical practice, complications such as septic bursitis, septic arthritis, necrotizing fasciitis, and tissue phlegmon have been reported during intra-articular or musculoskeletal injections [8]. Therefore, strict procedures must be followed during US-guided injections to maintain hygiene and prevent infection.

However, there is considerable variation in opinions about aseptic techniques, and evidence supporting strict aseptic procedures is limited, often leading to the adoption of less rigorous ‘clean’ techniques [9]. Although US probes used in guided interventional procedures are classified as semi-critical instruments requiring high-level disinfection according to the Spaulding classification [10,11,12], there is ongoing debate about the optimal approach to infection prevention. Some studies strongly recommend using high-level disinfectants, sterile gels, and sterile probe covers for maximum infection prevention [13,14,15]. Meanwhile, implementings all these sterile procedures in clinical practice has been criticized as potentially impractical due to time constraints, cost considerations, and overall efficiency [16,17].

Previous literature on US-guided injections has primarily focused on identifying treatment points and proper positioning of the probe and needle [18,19,20]. Existing guidelines on interventional US typically provide comprehensive information about general US device usage, covering various procedures such as diagnosis, puncture, and biopsy. While the British Medical Ultrasound Society (BMUS) guideline specifically addresses US-guided musculoskeletal injections, most guidelines provide limited guidance on detailed protocols specifically tailored for injection procedures [9,11,13,21]. Moreover, while these guidelines address probe reprocessing, probe covers, and US gel usage, they provide minimal guidance on skin disinfection procedures, which are crucial for injection safety.

Given the lack of detailed procedural guidance identified in previous literature and existing guidelines, there is a clear need to systematically map the hygiene and safety practices currently applied in US-guided injections. Therefore, this scoping review aims to achieve two primary objectives: first, to identify existing knowledge gaps regarding hygiene and safety protocols in US-guided procedures, and second, to systematically outline reported procedural techniques and analyze how US utilization can enhance the safety and accuracy of injection practices, using a scoping review method.

The hygiene and safety procedures outlined in this study can provide a foundation that can be developed into standardized protocols through expert consensus (Delphi method), ultimately helping healthcare professionals, including KMDs, perform US-guided injections more safely.

## 2. Methods

We conducted a scoping review to systematically investigate hygiene and safety procedures for US-guided injections, aiming to provide implications for future clinical practice and research. The review was performed following the methodological guidance provided by the Joanna Briggs Institute [22] and was reported according to the Preferred Reporting Items for Systematic Reviews and Meta-Analyses extension for Scoping Reviews (PRISMA-ScR) checklist [23] (Checklist S1). The review protocol was registered on the Open Science Framework (OSF) [https://osf.io/tv5p6/ (accessed on 5 March 2025)].

### 2.1. Review Questions

As mentioned above, the purpose of this study was to suggest safety guidelines that practitioners can follow for each step of the US-guided injection procedure. To clarify broad questions, our research questions were set as follows: (1) What are the skin and probe disinfection procedures being used? (2) What coupling agents are used for transmission? (3) What US-based methods are used to improve the safety and accuracy of the procedure? (4) What is currently known about US-guided injection procedures? and (5) What research gaps can be identified?

### 2.2. Search Strategy

We conducted a comprehensive search across two English databases (MEDLINE via PubMed, Cochrane Central Register of Controlled Trials) and two Korean databases (ScienceON, Koreanstudies Information Service System). The search strategy combined key terms including “ultrasonography”, “sonography”, “injection”, and “clinical trial” using appropriate Boolean operators (“AND”, “OR”) (Supplementary Table S1). Additionally, a manual search was carried out by investigating the references of the selected articles. Database searches covering publications from inception to November 2023 were executed between 8 and 11 November 2023. Duplicates were removed using EndNote 21.

### 2.3. Inclusion and Exclusion Criteria

#### 2.3.1. Inclusion Criteria

The selection criteria were established through research team consensus (Table 1). We primarily included clinical studies of US-guided injections or pharmacopuncture (herbal injection) performed on human subjects. Clinical study designs such as RCTs, non-randomized controlled trials, before-and-after studies, and non-comparative studies were included. Participants were human subjects undergoing musculoskeletal interventions. Studies published in English and Korean were considered eligible.

Given our aim to comprehensively map hygiene and safety practices in US-guided procedures, two key procedural aspects were required for inclusion. First, we selected papers that described pre-procedure skin disinfection, which is the most basic infection prevention process and a common practice in hospitals [24], since we aimed to synthesize current disinfection methods. Second, only studies that provided detailed information about probe placement and needle manipulation techniques were included to clarify the coordination procedure based on condition and target site [25,26].

#### 2.3.2. Exclusion Criteria

Reviews, meta-analyses, and letters were excluded. Considering the primary application of US-guided pharmacopuncture in musculoskeletal conditions by KMDs [5], we excluded non-musculoskeletal sites such as endovascular and peritumoral applications.

### 2.4. Study Screening and Selection

All studies identified through database searches were first screened by reviewing their titles and abstracts to assess eligibility based on the predefined inclusion and exclusion criteria. Full texts of potentially relevant studies were then retrieved and reviewed for detailed evaluation. Studies were excluded if they did not meet the selection criteria or if the full text was unavailable for review. Additionally, the reference lists of the included articles were manually searched to identify any additional eligible studies. Two independent reviewers (Y.K. and G.J.) conducted the screening and selection processes separately, and any disagreements were resolved through discussion with a third reviewer (E.C.).

### 2.5. Data Extraction and Data Charting

Before the extraction of the data, the research team held a meeting to determine the specific items to collect. These items included author, publication year, study design, participants (including disease or symptom), intervention and control groups, outcomes, and the procedure details. The procedure details encompassed preliminary US scanning, whether sterile gloves were used, the materials and methods for disinfecting the procedure site, and the types of US coupling agent. The study designs of the included studies were categorized based on the algorithm for classifying quantitative study designs of the National Institute for Health and Care Excellence [27]. A data recording form was created using Microsoft Excel 2016. We implemented pilot data extraction from five studies and checked and revised the extraction methods to ensure that they matched the research questions and purposes [28,29]. Afterwards, two researchers (Y.K., G.J.) independently extracted data, and any discrepancies were discussed and resolved with the involvement of a third reviewer (E.C.). All extracted data were organized in a structured format to facilitate narrative synthesis and thematic analysis.

### 2.6. Data Analysis and Presentation of Results

The results were analyzed and presented in four main sections. First, we summarized the general characteristics of the included studies, including publication trends and study designs. Second, procedural data were extracted using a predefined form and categorized based on their timing [before, during, after the injection] and purpose [hygiene, safety, accuracy]. Hygiene procedures were further sub-categorized into probe disinfection, use of probe cover, and skin disinfection. Third, we examined the types and applications of US coupling agents. Finally, we identified gaps between reported practices and recommended procedures, as well as current research gaps in the literature.

To identify these gaps, we conducted a comparative analysis between the reported procedural elements—such as skin disinfection methods, use and type of transducer covers, application of US gel, and hand hygiene—and the corresponding recommendations outlined in four major guidelines: BMUS [9], Society of Diagnostic Medical Sonography (SDMS) [13], European Federation of Societies for Ultrasound in Medicine and Biology (EFSUMB) [11], and German Society of Ultrasound in Medicine (DEGUM) [21]. A gap was determined to exist when a study either omitted a key procedural element recommended by these guidelines or described a method that differed in sterility level, disinfection agent, or application technique from guideline-based standards.

The findings were presented using both narrative synthesis and descriptive statistics, supported by tables and figures where appropriate.

## 3. Results

### 3.1. Selection of Studies

**Figure 1:**
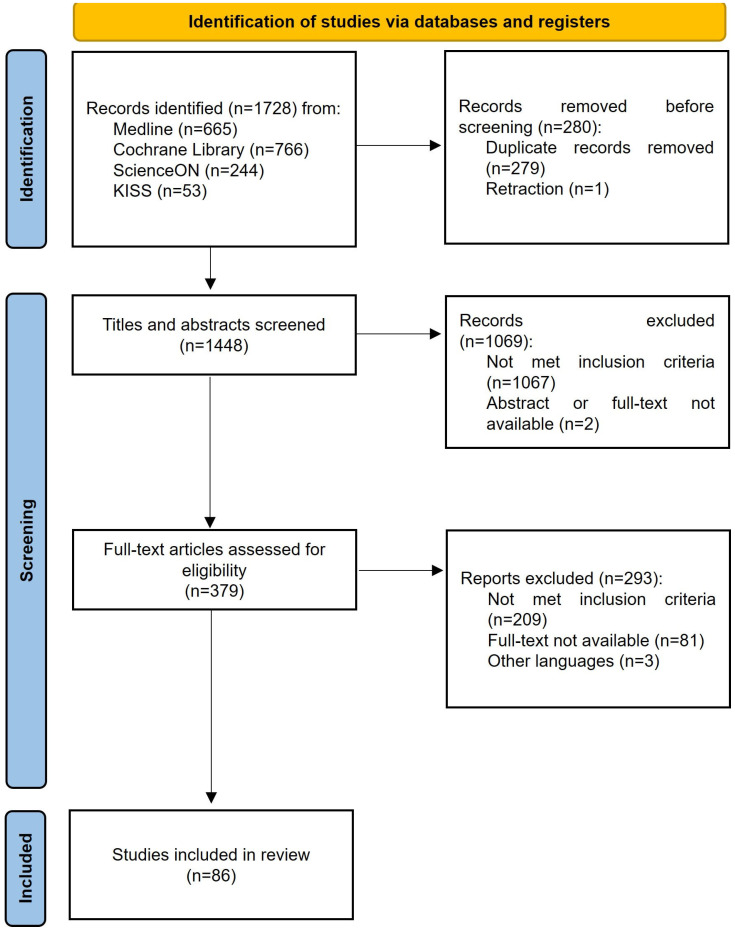
PRISMA flowchart for the study selection process. KISS, Koreanstudies Information Service System.

The search yielded a total of 1728 studies. After removing duplicates and retractions, 1448 studies were screened based on their titles and abstracts. Full texts of 379 studies were examined. Finally, 86 articles were selected for this review (Figure 1).

### 3.2. Characteristics of the Selected Studies

**Figure 2:**
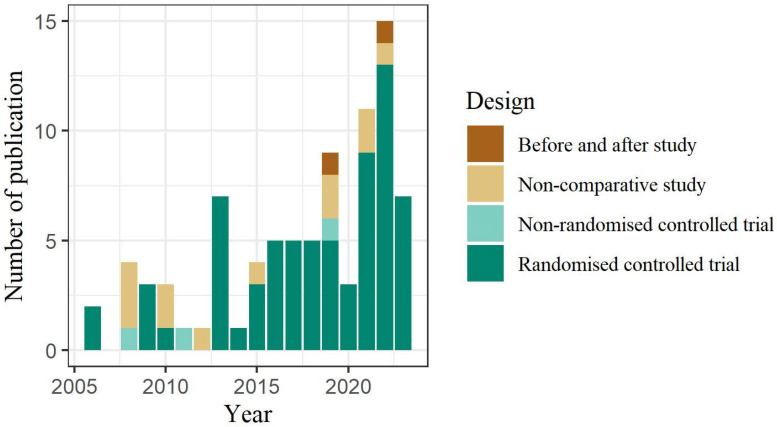
Study design of the selected studies by publication year.

The publication year of the selected studies varied between 2006 and 2023, and 72 (83.72%) studies were published after 2013. Information on the study designs by publication year is presented in Figure 2. Of the 86 articles, 69 (80.23%) were randomized controlled trials, 12 (13.95%) were non-comparative studies, 3 (3.49%) were non-randomized controlled trials, and 2 (2.33%) were before and after studies.

Based on the 11th revision of the International Classification of Diseases, ‘Diseases of the musculoskeletal system or connective tissue’ were the most common (59; 68.60%) in the selected studies, followed by ‘Diseases of the nervous system’ (16; 18.60%) (Table S2). In terms of the solutions used for US-guided injections, there were 82 medications from conventional medicine and 4 pharmacopuncture medications from KM (three Soyeom and one essential bee venom). Detailed information of the studies is provided in Table S3 (see Supplementary Materials), including types of medication, treatment site, syringe details (thickness and length), treatment dose, duration of treatment, intervention and control group information, outcome measures, and injection methods.

### 3.3. Procedures for US-Guided Injection in the Literature

The procedures reported in the procedures reported in the included studies were categorized as before, during, and after injection (Table 2). Additional procedural requirements specific to US-guided injections, compared to conventional blind injections, were classified into three categories: procedures for maintaining skin and probe hygiene, US coupling agent, and US-based methods used to enhance safety and accuracy.

#### 3.3.1. Procedures for Maintaining Skin and Probe Hygiene

The main procedures used for preventing infections are presented in Table 3. The procedures related to the probe setup included “disinfect the probe” (5.81%) and “affix the sterilized probe cover” (24; 27.91%). The materials used for disinfecting probe included chlorhexidine, a 45% solution of didecyldimethylammonium chloride, povidone–iodine solution, and 70% alcohol. The sterile probe covers included sterile sheaths, sterilized gloves, the Steri-Drape (3M Health Care), sterile transparent field dressing, and a sterile camera sleeve. All reviewed papers included skin disinfection procedures, with iodophors (18; 20.93%), alcohol (10; 11.63%), chlorhexidine (4; 4.65%), and benzalkonium chloride 0.25% (1; 1.16%) being the reported disinfectants.

#### 3.3.2. US Coupling Agent

Of the 23 articles (26.74%) that described the procedure for applying solutions for US transmission, 17 articles (19.77%) utilized an agent either with sterilizing or disinfecting properties. The materials used included sterile US gel (15; 17.44%), povidone–iodine solution (1; 1.16%), and an aqueous solution containing 85% ethanol (1; 1.16%). Six papers (6.98%) reported the application of unsterilized gel (Table 4).

**Table 3:** Procedures for maintaining probe and skin hygiene (N = 86).

Category	Procedure	Materials Used	Frequency	Reference
SProbe disinfection	Disinfect the probe	chlorhexidine, solution of didecyldiethylammonium chloride (DDAC) 0.45% ^a^, solution of povidone–iodine, 70% alcohol	5 (5.81%)	[30,31,32,33], [34] ^a^
	Not specified or inferred from images		81 (94.19%)	
Use of probe cover	Affix the sterilized probe cover	sterile barrier/sheath/cover/barrier/wrapping/film, sterilized gloves, sterile surgical drape, sterile transparent field dressing, sterile camera sleeve	24 (27.91%)	[30,35,36,37,38,39,40,41,42,43,44,45,46,47,48,49,50,51,52,53,54,55,56,57]
	Not affixed with the sterilized probe cover		9 (10.47%)	[31,32,34,58,59,60,61,62,63]
	Not specified or inferred from images		53 (61.63%)	
Skin disinfection	Disinfect with alcohol	alcohol swap, alcohol, 70% alcohol, aqueous solution containing ethanol 85% vol, isopropyl alcohol solution, alcohol-based disinfection solution	10 (11.63%)	[48,58,64,65,66,67,68,69,70,71]
	Disinfect with iodophors	povidone–iodine, a solution of iodopovidone 10%, 10% polyvidone–iodine, betadine, type II mucosal iodine	18 (20.93%)	[30,34,35,45,46,54,57,72,73,74,75,76,77,78,79,80,81,82] ^b^
	Disinfect with chlorhexidine	chlorhexidine, chlorhexidine 0.5% solution	4 (4.65%)	[33,42,50,83]
	Disinfect with alcohol and povidone–iodine solution	70% alcohol and povidone–iodine solution	2 (2.33%)	[32]
	Disinfect with chlorhexidine and alcohol solution	ChloraPrep, chlorhexidine and isopropyl alcohol solution, 2% chlorhexidine gluconate and 70% isopropyl alcohol, chlorhexidine alcohol 0.5%	4 (4.65%)	[62,84,85,86]
	Disinfect with benzalkonium chloride	benzalkonium chloride 0.25%	1 (1.16%)	[34] ^c^
	Disinfect with antiseptic (not specified)		50 (58.14%)	

Data are presented as frequencies and percentages (%). ^a^ Cleaned for at least two minutes to ensure complete sterilization. ^b^ Five applications of betadine, skin disinfectant, or an equivalent product for allergic patients. ^c^ Benzalkonium chloride 0.25% when allergy to iodine was reported by the patient.

**Table 4:** Coupling agent used for US-guided injection (N = 86).

Category	Procedure	Materials Specified	Frequency	Reference
Application of the US coupling agent to the probe	Apply nonsterile US gel		6 (6.98%)	[35,48,50,54,57,58]
	Apply sterile US gel	sterile gel/sterile ultrasound gel, sterile lubrication packets, sterile lubricant contact gel	15 (17.44%)	[32,34,37,38,39,42,44,47,52,56,66,83,84,87,88]
	Apply povidone–iodine solution		1 (1.16%)	[30]
	Apply 85% ethanol solution		1 (1.16%)	[67]
	Not specified or inferred from images		63 (73.26%)	

Data are presented as frequencies and percentages (%). Non-sterile US gel inside the sterile glove, sterile us gel between the probe and the skin.

#### 3.3.3. Comprehensive Overview of Infection Prevention Procedures

Integrated analysis of the aforementioned disinfection methods is presented in Table S4 (see Supplementary Materials). Two studies utilized identical disinfectants for both probe and skin preparation [31,33], with one specifically employing chlorhexidine [33]. Another study reported using an 85% ethanol solution as both the coupling agent and skin disinfectant [67]. One study described a protocol where povidone–iodine solution was used for initial disinfection of both probe and skin, followed by its reapplication for US transmission after complete drying [30]. Out of the six papers (6.98%) that used a non-sterilized solution, five reported the application of a non-sterile gel, with a sterile probe cover placed over it [35,48,50,54,57]. One study indicated that the skin at the treatment site was disinfected and that efforts were made to prevent direct contact between the probe and the treatment area [58].

#### 3.3.4. US-Based Methods to Enhance Safety and Accuracy

Studies reported various methods to enhance accuracy, including “check the swelling of the joint capsule/bursa/sheath using US” [32,50,60,63,64,67,89], “check that the surrounding tissue is free of swelling on US” [46], “check the needle location by using shading as well as the flow of the drug while injecting a small amount of medication” [31,67,73], and “adjust the probe after inserting the needle to secure a clear view” [56]. Some studies used the anisotropy of the tendon to locate the target by flexing the joint before injection when the target was a tendon [48].

In 17 (19.77%) of the selected studies, preliminary US scan was performed before the real-time US injection. Preliminary scan refers to the use of US imaging before the procedure. This procedure enhances accuracy and safety in US-guided injections by accurately identifying the correct position of the target, optimal needle entry point and angle, needle depth, and any abnormalities [90].

#### 3.3.5. Gaps Between Reported Practices and Recommended Guidelines

Several discrepancies were found between the reported infection prevention practices and recommended protocols. Some studies reported using sterilized probe covers without concurrent probe disinfection, while others documented the use of non-sterile gel beneath sterile covers. This practice raises concerns, as evidence suggests that probe covers may tear during procedures, necessitating both probe disinfection and sterile coupling agents regardless of cover usage [11,91,92]. When non-sterile US gel is unavoidable, current guidelines strongly advocate for single-use gel packets rather than multi-use containers to minimize contamination risk [91]. One study’s approach of maintaining probe-skin separation following skin disinfection [58] contradicts established recommendations, as this method poses risks of inadvertent contamination of both the procedure site and needle [7,21].

## 4. Discussion

### 4.1. Summary of Findings

US-guided injections are a complex process that requires simultaneous manipulation of the probe and needle under hygiene conditions. This study systematically reviewed the methods used to ensure hygiene and safety during US-guided interventions, as reported in published clinical studies. Although international guidelines—including those from BMUS [9], SDMS [13], EFSUMB [11], and DEGUM [21]—consistently emphasize core hygiene principles for US-guided procedures, our review revealed that actual implementation in clinical practice remains inconsistent. Most guidelines emphasize the use of sterile, disposable transducer covers; skin antisepsis and hand hygiene consistent with high-level disinfection protocols; and sterile, single-use US gel. However, only 5.81% of the studies reported any transducer-related disinfection procedures, and just 27.91% documented the use of sterile probe covers. Skin disinfection methods also varied considerably, with iodophors (20.93%) and alcohol-based solutions (11.63%) being the most frequently reported. Moreover, while 26.74% of studies described the application of US coupling agents, fewer than 20% explicitly stated that sterile agents were used. These findings highlight substantial variation in practice and indicate that key infection control elements remain under-implemented or insufficiently reported.

Notably, most existing guidelines provide general recommendations for infection control in US-guided interventions but are not tailored specifically to musculoskeletal applications. This lack of context-specific guidance may partly explain the observed variation and inconsistency in hygiene practices across the reviewed studies. Nevertheless, our review systematically compared the reported procedures with major guidelines [9,11,13,21], offering a structured overview of how current practices align—or diverge—from established standards. By mapping this gap, the present study contributes foundational evidence that may support the development of standardized, consensus-based protocols for musculoskeletal US-guided injections, ultimately improving safety and reproducibility in clinical practice.

In addition to the inconsistencies between reported practices and guideline recommendations, the review also revealed notable research gaps in the literature. Specifically, few studies addressed operator hand hygiene in detail, post-procedural transducer processing, or the sequence of infection prevention steps, which were often either briefly mentioned or entirely omitted. These findings underscore the need for further research to establish standardized reporting practices and evidence-based protocols in musculoskeletal US-guided interventions.

### 4.2. Debates on Hygiene Protocols During US-Guided Procedures

Unlike conventional injections, US-guided procedures present unique challenges for infection control. The procedure requires simultaneous manipulation of both the US probe and syringe in close proximity, while the use of coupling agents necessary for acoustic impedance matching creates additional risks for contamination of the treatment site [6,7]. These infection control challenges can lead to serious complications such as septic arthritis, bursitis, and tissue phlegmon if not properly addressed [8]. These additional complexities necessitate specific infection control protocols beyond those used in traditional injection procedures. Therefore, we focused our investigation on hygiene-related procedures to ensure patient safety during US-guided interventions.

Current debates regarding appropriate hygiene protocols center around several key aspects. First, the choice of skin disinfectants remains controversial. Studies suggest that chlorhexidine–alcohol solutions may be superior to povidone–iodine or alcohol alone in preventing infections [93,94]. However, our review found that iodophors (20.93%) and alcohol-based solutions (11.63%) were the most used disinfectants, while chlorhexidine was reported in only 4.65% of studies. Furthermore, some practitioners advocate for a two-step cleaning procedure using colored followed by colorless antiseptics [17], but this approach was rarely documented in our review. These differences suggest the need for further research and standardized protocols for effective disinfection methods in interventional US.

Second, there are differing views on probe protection methods. Some argue for using sterile gels and probe covers to prevent equipment damage, as wiping the probe directly with disinfectants can degrade its quality [95,96,97]. Others consider using sterile gels or liquids along with sterile sheaths, condoms, or gloves or non-sterile gel-filled condoms or gloves unnecessarily costly [17]. This viewpoint is supported by studies demonstrating no infection when the US probe was simply cleaned with an antiseptic sterile solution during the procedure [98,99,100]. This is reflected in our finding that only a small proportion of studies reported probe disinfection or cover use.

Third, the use of sterile equipment varies widely in clinical practice. Less than 30% of selected studies reported using sterile gels or probe covers. However, some argue that sterile probe covers should be used to maintain the highest standards of sterility [101,102]. In some articles, the protocols were adjusted based on procedure duration, using minimal sterile materials for brief treatments while implementing more rigorous sterility measures for longer or major procedures [11,99,100]. Intra-articular procedures typically demand stricter sterilization protocols, using povidone–iodine or alcohol for skin disinfection as well as sterile probe covers and gels [103].

Despite these debates surrounding hygiene protocols during US-guided injections, it is crucial to prioritize patient safety by reducing the risk of infection. When performing a safe US-guided injection, it is important to disinfect the skin; use a probe cover, sterile US gel, or disinfectants as a coupling agent; and re-disinfect and bandage the treatment area after the procedure. These measures were found to be performed relatively frequently in our study and should be prioritized.

### 4.3. Limitations of This Study

This study has several limitations. First, our selection criteria emphasized skin disinfection procedures, potentially underrepresenting other procedures in US-guided injections. Studies that did not explicitly describe skin disinfection protocols were excluded, which may have omitted relevant information from other sources. Second, our analysis focused primarily on infection prevention measures, leaving gaps in our understanding of broader infection control practices. For instance, while probe reprocessing involves multiple steps including cleaning, disinfection, sterilization, and storage, our review captured only the immediate pre-procedural aspects of probe handling. Third, this review may not fully capture the actual clinical practices compared to survey-based research methods. Additionally, formal quality assessment of the included studies was not conducted. This approach is consistent with established scoping review methodology (e.g., JBI, PRISMA-ScR), which prioritizes mapping the extent and nature of evidence rather than synthesizing findings based on study quality appraisal. Despite these limitations, our study provides valuable insights into hygiene and safety procedures documented in clinical studies, contributing to the groundwork for developing standardized protocols for US-guided injections.

### 4.4. Practical Implications for Clinical Decision Making

While this scoping review highlights significant variation in reported hygiene practices during US-guided injections, it provides the following practical implications for clinicians aiming to enhance procedural safety. First, consistent skin disinfection using an appropriate agent is fundamental and crucial; although some studies suggest the superiority of chlorhexidine-alcohol solutions, iodophors or alcohol-based solutions remain widely used. Second, the use of sterile probe covers and sterile coupling agents is recommended, especially for procedures requiring strict asepsis, such as intra-articular injections. However, given the low reported usage rates and practical constraints related to cost and time, clinicians must balance these recommendations against feasibility in their specific context. If non-sterile gel is used, employing single-use packets may help reduce contamination risk. Third, regardless of probe cover use, appropriate probe disinfection and reprocessing according to guidelines are essential, considering the risk of contamination from factors like micro-perforations. Fourth, utilizing a preliminary US scan before the procedure can contribute to enhancing procedural accuracy and safety. These suggestions, based on the analysis of the current literature, can help guide clinical decision making pending the development of more definitive, context-specific guidelines.

### 4.5. Research and Guideline Development Implications

Our review revealed substantial gaps in the current literature regarding hygiene and safety procedures in US-guided injections, which highlight several important directions for future investigation and guideline development. First, a significant number of studies lacked sufficient methodological detail; 61% (166/272) of potentially relevant articles were excluded due to inadequate or non-specific descriptions of skin disinfection protocols. Even among the included studies, more than half provided incomplete information regarding probe and skin hygiene, such as antiseptic types, probe cover utilization, and coupling agents for US transmission. Detailed considerations or procedural sequences, including precise timing and extent of skin disinfection and recommended waiting periods post-disinfection, were largely absent. Additionally, post-procedural probe reprocessing (e.g., cleaning, disinfection, and storage) was also largely omitted.

To address these deficiencies, future clinical guidelines and protocols should comprehensively address hygiene and safety procedures, including detailed considerations of skin antisepsis, operator hand hygiene, the sequence of infection control steps, and post-procedural disinfection practices. Incorporating these components into standardized recommendations will enhance clarity and promote more consistent implementation across clinical settings.

However, while current guidelines emphasize strict aseptic protocols, their feasibility—particularly for musculoskeletal US-guided interventions—remains uncertain. Future research should evaluate which combinations of hygiene practices (e.g., type of disinfectant, use of probe covers, gel sterility) are both safe and feasible in real-world settings. In addition to refining procedural elements, studies should assess key outcomes such as infection rates, cost-effectiveness, and provider adherence. Expert consensus methods (e.g., Delphi panels) and pragmatic trials can further support the development of evidence-based, scalable protocols tailored to various clinical environments.

## 5. Conclusions

This scoping review identified key procedures for ensuring safety and hygiene in US-guided injections, encompassing pre-procedural preparation, infection prevention during the procedure, and post-procedural care. While current practices vary considerably, particularly regarding probe disinfection, probe cover use, and coupling agent selection, our findings emphasize the importance of detailed and sequential prevention protocols specific to US-guided procedures. The notable gaps in documentation and standardization of hygiene procedures suggest the need for further research to develop evidence-based protocols tailored to different anatomical sites and types of injections. This work provides a foundation for developing safety guidelines through expert consensus and real-world implementation studies, ultimately enhancing the safety and reproducibility of US-guided procedures across various healthcare specialties, including Korean Medicine.

## Figures and Tables

**Table 1:** Inclusion and exclusion criteria for this scoping review.

Criteria	Inclusion	Exclusion
Study design	Clinical study	Review, meta-analysis, response to letter
Participants	Human (no limitations)	Non-human (cadavers, animals)
Target site	Musculoskeletal interventions: Muscles Skin Ligaments Tendons Nerves Perivascular tissues Intra-articular space	Non-musculoskeletal sites: Endovascular Lacrimal Peritumoral Special procedures: Transvaginal US Transrectal US Endoscopic US
Intervention	(1) US-guided injection (2) US-guided pharmacopuncture (3) Combined interventions where injection was administered independently: Local anesthetic injection (lidocane, bupivacane) without nerve block Peritendinous or intratendinous injections near neovascularization Intra-articular injection with concurrent fluid aspiration Calcific tendon treatment combining injection with needle fragmentation Post-nerve block injections	(1) Non-injection procedures Acupotomy Percutaneous lavage Cryoneurolysis Nerve ablation Aspiration only Fragmentation Other US-guided percutaneous treatments (2) Neural blockade procedures Nerve blocks Hematoma blocks Catheterization blocks Simplified adductor canal blocks (e.g., “SAC block”) (3) Sequential procedures Injection following puncture Injection after fragmentation
Procedure description	Must include: Skin disinfection procedures	Excluded if lacking: Detailed probe placement (anatomical location, angle to structures, direction of approach) Detailed needle insertion information (e.g., in-plane or out-of-plane)
Control	No limitation	
Outcome	No limitation	

US, ultrasound.

**Table 2:** Reported procedures for US-guided injection in the selected studies (N = 86).

Category		Procedure	Frequency
Before the injection			
	Informed consent	Explain the procedure and precautions and obtain consent	10 (11.63%)
	Allergic reaction test	Test for allergic reactions prior to the procedure	1 (1.16%)
	Probe disinfection	Disinfect the probe	5 (5.81%)
	Use of probe cover	Affix the sterilized probe cover	24 (27.91%)
		Omit affixing the sterilized probe cover	9 (10.47%)
	Application of the US coupling agent to the probe	Apply nonsterile ultrasound gel	6 (6.98%)
		Apply sterile ultrasound gel	15 (17.44%)
		Apply povidone–iodine solution	1 (1.16%)
		Apply 85% ethanol solution	1 (1.16%)
	Putting on sterile gloves	Wear sterile gloves	5 (5.81%)
	Draping the treatment area	Cover the treatment area with disinfectant cloths/drapes	14 (16.28%)
	Preliminary US scan	Perform a preliminary US scan	17 (19.77%)
	Local anesthesia	Locally anesthetize the area to be treated	24 (27.91%)
During the injection			
	Skin disinfection	Disinfect with alcohol	10 (11.63%)
		Disinfect with iodophors	18 (20.93%)
		Disinfect with chlorhexidine	4 (4.65%)
		Disinfect with alcohol and povidone–iodine solution	2 (2.33%)
		Disinfect with chlorhexidine and alcohol solution	4 (4.65%)
		Disinfect with benzalkonium chloride	1 (1.16%)
		Disinfect with antiseptic (not specified)	50 (58.14%)
	Regurging	Regurge the syringe	1 (1.16%)
After the injection			
	Post-procedure care	Re-disinfect the injection site	12 (13.95%)
		Apply compression hemostasis to the procedure site	3 (3.49%)
		Apply a bandage to the injection site	13 (15.12%)
	Post-procedure monitoring	Allow the patient to rest and monitor for any side effects	13 (15.12%)
	Post-procedure instructions for patients	Instruct the patient to prevent contamination or contact with water	3 (3.49%)
		Instruct the patient to promptly report any side effects	7 (8.14%)
		Instruct the patient to avoid excessive movement and pressure on the treated area	22 (25.58%)
		Instruct the patient to apply ice pack in case of pain or discomfort	12 (13.95%)
		Instruct the patient to take medication in case of pain or discomfort	14 (16.28%)

Data are presented as frequencies and percentages (%).

## Data Availability

The original contributions presented in this study are included in the article/Supplementary Materials. Further inquiries can be directed to the corresponding authors.

## Supplementary Materials

The following supporting information can be downloaded at https://www.mdpi.com/article/10.3390/healthcare13101165/s1. Checklist S1. Preferred Reporting Items for Systematic reviews and Meta-Analyses extension for Scoping Reviews (PRISMA-ScR) checklist. Table S1: Search strategies. Table S2: The diseases of participants classified by the ICD-11. Table S3: Detailed information of the studies. Table S4: Integrated analysis of aforementioned disinfection methods.

## Abbreviations

The following abbreviations are used in this manuscript:

DDAC	Didecyldiethylammonium chloride
US	Ultrasound
KISS	Koreanstudies Information Service System
KMD	Korean Medicine doctor
