# Supplementary material for: A Scoping Review of Clinical Studies on Procedures of Ultrasound-Guided Injection to Ensure Hygiene and Safety
Source: Healthcare (Basel). 2025 May 16;13(10):1165. doi: 10.3390/healthcare13101165 (PMC12110851; doi:10.3390/healthcare13101165)
Supplement: Supplementary file 1 [file healthcare-13-01165-s001.zip › Table S4.pdf]

Table S4. Integrated analysis of aforementioned disinfection methods

Year	Author	Title	Probe disinfection	Probe cover	Skin disinfection	US scanning agent
2006	Kim et al.	The Usefulness of Ultrasonographyguided Injection Comparing to Blind Injection in Patients with Chronic Subdeltoid Bursitis	NA	Sterilized gloves	povidone-iodine	NA
2021	Kim et al.	The Effectiveness of Ultrasound-Guided Soyeom Pharmacopuncture Therapy at Acromioclavicular Joint of Shoulder in Patients with Anterior Shoulder Pain: A Retrospective Study	NA	X (Photo)	alcohol swap	NA
2019	Kim et al.	A Comparative Study on the Pain and Treatment Satisfaction between Korean Medical Treatment Combined with Ultrasound Guided Soyeom Pharmacopuncture Therapy in Thoracic Paravertebral Space and Non-Guided Soyeom Pharmacopuncture Therapy on Patients with Ribs Fracture: A Retrospective Study	NA	X (Photo)	alcohol swap	NA
2008	Nam et al.	Ultrasound-Guided Proliferative and Local Steroid Injection for Subacromial Bursitis	NA	NA	povidone-iodine	NA
2008	Nam et al.	Injection Therapy for Calcific Tendinitis of Shoulder Under the Sonographic Guidance	NA	NA	povidone-iodine	NA
2008	Moon et al.	Blind and Ultrasonography-guided Injection Therapy for Calcific Tendinitis of Supraspinatus	NA	NA	povidone-iodine	NA
2010	Moon et al.	The ultrasound-guided injection of prolotherapy and steroid mixture in patients with adhesive capsulitis	NA	NA	povidone-iodine	NA
2010	Moon et al.	The Effectiveness of Prolotherapy Postoperative Rotator Cuff Tear	NA	X (Photo)	povidone iodine and alcohol	NA
2015	Pak et al.	The Effectiveness of Ultrasonography-guided Injection at Infraspinatus of Shoulder in Patients with Posterior Shoulder Pain Induced Horizontal Adduction Test	NA	X (Photo)	NS (povidone-iodine; Photo)	NA
2019	Ahn et al.	The Effectiveness of Ultrasound-Guided Essential Bee Venom Pharmacopuncture Combined with Integrative Korean Medical Treatment for Rib Fracture: A Case Study	potadine solution [c]	sterile surgical drape	potadine solution	potadine solution
2022	Yang et al.	Comparison of Ultrasound Guided Soyeom Pharmacopuncture Therapy Effect and Unguided Soyeom Pharmacopuncture Therapy Effect on Cervical Facet Joint of Acute Cervical Pain Patient Caused by Traffic Accidents: A Retrospective Study	NA	NA	alcohol	NA
2009	Yoon et al.	Ultrasonography-guided Subacromial Bursal Injection of Corticosteroid: A Comparative Study of Two Dose Regimens	NA	NA	NS	NA
2012	Lhee et al.	Accuracy and Clinical Outcomes of Ultrasound-guided Glenohumeral joint Injection: Acromioclavicular Approach in Patients with Adhesive Capsulitis	NA	X (Photo)	NS (povidone-iodine; Photo)	NS (Photo)
2008	Lim et al.	Ultrasound Guided Shoulder Joint Injection through Rotator Cuff Interval	NA	sanitized plastic cover	povidone iodine	NS (Photo)
2010	Cho et al.	Comparision of Blind Technique and Ultrasonography Guided Technique of Subacromial Subdeltoid Bursa Injection	NA	NA	alcohol	NA
2022	Ahadi et al.	Ultrasound-Guided vs. Blind Coccygeal Corticosteroid Injections for Chronic Coccydynia: A Randomized, Clinical Trial	NA	a sterile sheath	betadine	ultrasound gel
2022	Ahadi et al.	Comparison of the Effect of Ultrasound-Guided Injection of Botulinum Toxin Type A and Corticosteroid in the Treatment of Chronic Plantar Fasciitis: A Randomized Controlled Trial	NA	sterile barrier	NS (antiseptic)	NA
2022	Anwar et al.	Combined effectiveness of extracorporeal radial shockwave therapy and ultrasound-guided trigger point injection of lidocaine in upper trapezius myofascial pain syndrome	NA	NA	NS (local antiseptic)	NA
2020	Babaei-Ghazani et al.	Ultrasound-Guided Corticosteroid Injection in Carpal Tunnel Syndrome: Comparison Between Radial and Ulnar Approaches	NA	NA	NS (antiseptic)	NA
2019	Babaei-Ghazani et al.	Comparison of Ultrasound-Guided Local Ozone (O2-O3) Injection vs Corticosteroid Injection in the Treatment of Chronic Plantar Fasciitis: A Randomized Clinical Trial	NA	sterile barrier	NS (antiseptic)	sterile gel
2018	Babaei-Ghazani et al.	The effects of ultrasound-guided corticosteroid injection compared to oxygen–ozone (O2–O3) injection in patients with knee osteoarthritis: a randomized controlled trial	NA	sterile barrier	NS (antiseptic)	sterile gel
2018	Babaei-Ghazani et al.	Comparison Between Effectiveness of Ultrasound-Guided Corticosteroid Injection Above Versus Below the Median Nerve in Mild to Moderate Carpal Tunnel Syndrome: A Randomized Controlled Trial	NA	sterile barrier	NS (antiseptic)	sterile gel
2018	Chen et al.	Effectiveness of ultrasound-guided vs direct approach corticosteroid injections for carpal tunnel syndrome: A double-blind randomized controlled trial	NA	NA	NS	NA
2016	Cole et al.	Ultrasound-Guided Versus Blind Subacromial Corticosteroid Injections for Subacromial Impingement Syndrome: A Randomized, Double-Blind Clinical Trial	NA	X	alcohol	nonsterile gel
2022	Creuzé et al.	Novel Use of Botulinum Toxin in Long-Standing Adductor-Related Groin Pain: A Case Series	NA	NA	70% alcohol	NA
2016	Di Sante et al.	Intra-articular hyaluronic acid vs platelet-rich plasma in the treatment of hip osteoarthritis	NA	X (Photo)	NS (povidone-iodine; Photo)	NA
2023	Ebadi et al.	Extracorporeal shockwaves therapy versus corticosteroid injection for the treatment of non-calcific rotator cuff tendinopathies: a randomized trial	NA	sterile barrier	NS (antiseptic)	NA
2009	Ekeberg et al.	Subacromial ultrasound guided or systemic steroid injection for rotator cuff disease: randomised double blind study	NA	NA	alcohol	sterile gel
2021	Emami Razavi et al.	Short-term Efficacy of Ultrasonographic Guidance for Intra-articular Corticosteroid Injection in Hallux Rigidus: A Single-Blind Randomized Controlled Trial	NA	NS	povidone-iodine	NA
2017	Eslamian et al.	A Randomized Prospective Comparison of Ultrasound-Guided and Landmark-Guided Steroid Injections for Carpal Tunnel Syndrome	NA	a sterile US transducer cover	NS (antiseptic)	NA
2023	Farrow et al.	Ultrasound-Guided Trigger Point Injections for the Treatment of Neck and Back Pain in the Emergency Department: A Randomized Trial	NA	a sterile transparent field dressing	chlorhexidine	sterile lubrication packets
2019	Frizziero et al.	Efficacy of ultrasound-guided hyaluronic acid injections in achilles and patellar tendinopathies: a prospective multicentric clinical trial	NA	NA	NS (povidone-iodine; Photo)	NA

2017	Gulabi et al.	USG-guided injection of corticosteroid for lateral epicondylitis does not improve clinical outcomes: a prospective randomised study	NA	sterile cover	NS	NA
2015	Haghighat et al.	Effectiveness of Blind & Ultrasound Guided Corticosteroid Injection in Impingement Syndrome	NS(disinfectant)	X	NS (disinfectant)	NA
2013	Hansen et al.	Surgery versus ultrasound-guided steroid injections for trigger finger disease: protocol of a randomized controlled trial	NA	NA	an aqueous solution containing ethanol 85% vol	an aqueous solution containing ethanol 85% vol
2016	Hazra et al.	Ultrasound versus fluoroscopy-guided caudal epidural steroid injection for the treatment of chronic low back pain with radiculopathy: A randomised, controlled clinical trial	NA	sterile transducer sheath	NS	sterile gel
2021	Hogaboom et al.	A pilot study to evaluate micro-fragmented adipose tissue injection under ultrasound guidance for the treatment of refractory rotator cuff disease in wheelchair users with spinal cord injury	NA	NA	chlorhexidine/isopropyl alcohol solution	sterile ultrasound gel
2022	Hou et al.	The Effectiveness of Ultrasound-Guided Subacromial-Subdeltoid Bursa Combined With Long Head of the Biceps Tendon Sheath Corticosteroid Injection for Hemiplegic Shoulder Pain: A Randomized Controlled Trial	NA	NA	NS	NA
2013	Hsieh et al.	Is ultrasound-guided injection more effective in chronic subacromial bursitis?	NA	NA	NS	NA
2021	Hsieh et al.	Comparison of the corticosteroid injection and hyaluronate in the treatment of chronic subacromial bursitis: A randomized controlled trial	NA	NA	NS	NA
2022	Hsieh et al.	Effects of Intra-articular Coinjections of Hyaluronic Acid and Hypertonic Dextrose on Knee Osteoarthritis: a Prospective, Randomized, Double-Blind Trial	NA	NA	NS	NA
2011	Huang et al.	Efficacy and safety of ultrasound-guided local injections of etanercept into entheses of ankylosing spondylitis patients with refractory Achilles enthesitis	NA	NA	iodophors	NA
2018	Jones et al.	A randomized, controlled study to evaluate the efficacy of intra-articular, autologous adipose tissue injections for the treatment of mild-to-moderate knee osteoarthritis compared to hyaluronic acid: a study protocol	NA	NA	NS	NA
2021	Jurgensmeier et al.	Intra-articular Injections of the Hip and Knee With Triamcinolone vs Ketorolac: A Randomized Controlled Trial	NA	NA	ChloraPrep	NA
2017	Karaahmet et al.	Comparing the effectiveness of ultrasound-guided versus blind steroid injection in the treatment of severe carpal tunnel syndrome	NA	X (Photo)	NS	NA
2021	Karabaş et al.	Effects of ultrasound guided leukocyte-rich platelet-rich plasma (LR-PRP) injection in patients with pes anserinus tendinobursitis	NA	NA	10 % polyvidone-iodine	NA
2020	Karkucak et al.	Comparison of clinical outcomes of ultrasonography-guided and blind local injections in facet syndrome: A 6-week randomized controlled trial	NA	X	NS (povidone-iodine; Photo)	NA
2023	Keshmiri et al.	Clinical Effectiveness of Ultrasound-Guided Biolaser Versus Ozone Therapy in Reducing Chronic Pain in Knee Osteoarthritis: a Three-Month Follow-Up Study	NA	NA	povidone-iodine	NA
2017	Kim et al.	Comparison Between Anterior and Posterior Approaches for Ultrasound-Guided Glenohumeral Steroid Injection in Primary Adhesive Capsulitis: A Randomized Controlled Trial	70% alcohol	X	70% alcohol and povidone-iodine solution	a thin layer of sterile ultrasonographic transmission gel
2009	Lee et al.	Randomized controlled trial for efficacy of intra-articular injection for adhesive capsulitis: ultrasonography-guided versus blind technique	NA	X	NS (povidone-iodine; Photo)	NA
2022	Lee et al.	Comparison of Extracorporeal Shock Wave Therapy and Ultrasound-Guided Shoulder Injection Therapy in Patients with Supraspinatus Tendinitis	NA	NA	alcohol swab	NA
2013	Loizides et al.	Ultrasound guided versus CT-controlled pararadicular injections in the lumbar spine: a prospective randomized clinical trial	NA	sterile cover	NS	sterile US gel
2016	Mellor et al.	Exercise and load modification versus corticosteroid injection versus 'wait and see' for persistent gluteus medius/minimus tendinopathy (the LEAP trial): a protocol for a randomised clinical trial	chlorhexidine	NA	chlorhexidine	NA
2021	Mezian et al.	Ultrasound-guided perineural vs. peritendinous corticosteroid injections in carpal tunnel syndrome: a randomized controlled trial	NA	sterile glove	alcohol-based disinfection solution	non-sterile us gel, sterile us gel[b]
2020	Michaut et al.	Management of acromioclavicular joint disease by manual therapy versus corticosteroid injections: the protocol of a non-inferiority study	NA	NA	Betadine, skin disinfectant, or an equivalent product for allergic patients [d]	NA
2015	Misirlioglu et al.	Piriformis syndrome: comparison of the effectiveness of local anesthetic and corticosteroid injections: a double-blinded, randomized controlled study	NA	X	NS (povidone-iodine; Photo)	NA
2014	Monto et al.	Platelet-rich plasma efficacy versus corticosteroid injection treatment for chronic severe plantar fasciitis	NA	X	2% chlorhexadine gluconate/70% isopropyl alcohol	NA
2022	Nouri et al.	Comparison between the effects of ultrasound guided intra-articular injections of platelet-rich plasma (PRP), high molecular weight hyaluronic acid, and their combination in hip osteoarthritis: a randomized clinical trial	NA	NS	NS	NA
2013	Obernauer et al.	Ultrasound-guided versus Computed Tomography-controlled facet joint injections in the middle and lower cervical spine: a prospective randomized clinical trial	NA	NA	NS	sterile US gel
2015	Orlandi et al.	Ultrasound-guided percutaneous injection to treat de Quervain's disease using three different techniques: a randomized controlled trial	solution of didecyldiethylammonium chloride 0.45%[a]	X	a solution of iodopovidone 10% (or benzalkonium chloride 0.25 % when allergy to iodine was reported by the patient)	sterile lubricant contact gel
2013	Park et al.	Treatment effects of ultrasound-guided capsular distension with hyaluronic acid in adhesive capsulitis of the shoulder	NA	X	NS (povidone-iodine; Photo)	NA

2022	Paskins et al.	Clinical effectiveness of one ultrasound guided intra-articular corticosteroid and local anaesthetic injection in addition to advice and education for hip osteoarthritis (HIT trial): single blind, parallel group, three arm, randomised controlled trial	NA	sterile sheath	chlorhexidine 0.5% solution	gel
2023	Plaikner et al.	Ultrasound-guided versus computed tomography-controlled periradicular injections of the first sacral nerve: a prospective randomized clinical trial	NA	sterile coverage	NS	NA
2023	Radwan et al.	Genicular nerve block in juvenile idiopathic arthritis: a randomized clinical trial	NA	NA	NS	NA
2022	Rajaei et al.	The impact of intra-sacroiliac joint methylprednisolone injection in the recovery of patients with spondyloarthropathy: a randomized controlled trial	NA	NA	NS	NA
2019	Riel et al.	Heavy-slow resistance training in addition to an ultrasound-guided corticosteroid injection for individuals with plantar fasciopathy: a feasibility study	NA	NA	chlorhexidine alcohol 0.5%	NA
2021	Roddy et al.	Optimising outcomes of exercise and corticosteroid injection in patients with subacromial pain (impingement) syndrome: a factorial randomised trial	NA	NA	chlorhexidine 0.5% solution	sterile gel
2023	Sabaghzadeh et al.	Efficacy of Platelet-Rich Plasma for Chronic Lateral Ankle Instability After Modified Broström-Gould surgery: A Randomized, Single-Blinded, Prospective Controlled Trial	NA	sterile barrier	NS	sterile gel
2013	Sabeti-Aschraf et al.	Intra-articular versus periarticular acromioclavicular joint injection: a multicenter, prospective, randomized, controlled trial	NA	NA	NS	NA
2018	Salman Roghani et al.	Different doses of steroid injection in elderly patients with carpal tunnel syndrome: a triple-blind, randomized, controlled trial	NA	NA	NS	NA
2022	Santiago et al.	Long-term comparison between blind and ultrasound-guided corticoid injections in Morton neuroma	NA	NA	NS	NA
2019	Scott et al.	Platelet-Rich Plasma for Patellar Tendinopathy: A Randomized Controlled Trial of Leukocyte-Rich PRP or Leukocyte-Poor PRP Versus Saline	NA	NA	NS	NA
2019	Senna et al.	Platelet-rich plasma in treatment of patients with idiopathic carpal tunnel syndrome	NA	NA	NS (antiseptic solution)	NA
2013	Stenhouse et al.	Do blood growth factors offer additional benefit in refractory lateral epicondylitis? A prospective, randomized pilot trial of dry needling as a stand-alone procedure versus dry needling and autologous conditioned plasma	NA	NA	NS (antiseptic solution)	NA
2021	Tantanatip et al.	Comparison of the Effects of Physiologic Saline Interfascial and Lidocaine Trigger Point Injections in Treatment of Myofascial Pain Syndrome: A Double-Blind Randomized Controlled Trial	NA	NA	NS (antiseptic agent)	NA
2021	Thepsoparn et al.	Comparison of a Platelet-Rich Plasma Injection and a Conventional Steroid Injection for Pain Relief and Functional Improvement of Partial Supraspinatus Tears	NA	sterile camera sleeve	NS	NA
2022	Tiwari et al.	An Ultrasound-Guided Interfascial Injection Approach Versus an Ultrasound-Assisted Nerve Stimulating Approach of Obturator Nerve Block: A Randomized Clinical Trial	NA	a sterile plastic cover	povidone-iodine solution	gel
2006	Tsai et al.	Plantar fasciitis treated with local steroid injection: comparison between sonographic and palpation guidance	NA	NA	povidone-iodin	NA
2019	Vahdatpour et al.	Carpal tunnel syndrome treatment using ultrasound-guided versus landmark-guided corticosteroid injection: a randomized prospective trial	NA	NA	NS	NA
2021	Vasudeva et al.	Efficacy of a local corticosteroid injection on pain, disability and radial nerve thickness in patients with lateral epicondylitis	NA	NA	NS (antiseptics)	NA
2017	Wang et al.	Short-term effect of ultrasound-guided low-molecular-weight hyaluronic acid injection on clinical outcomes and imaging changes in patients with rheumatoid arthritis of the ankle and foot joints. A randomized controlled pilot trial	NA	sterilized plastic covering	NS	NA
2022	Wheeler et al.	Autologous Blood Injection With Dry-Needling vs Dry-Needling Alone Treatment for Chronic Plantar Fasciitis: A Randomized Controlled Trial	NA	NA	NS	sterile gel
2016	Yang et al.	Ultrasound-guided Versus Fluoroscopy-controlled Lumbar Transforaminal Epidural Injections: A Prospective Randomized Clinical Trial	NA	sterile wrapping	NS	sterile US gel
2022	Yu et al.	Clinical Effects and Safety of the Use of Methylene Blue for the Treatment of Lumbar Facet Joint Syndrome	NA	sterile film	povidone iodine	coupling agent
2023	Zhang et al.	Comparative study of ultrasonic-guided betamethasone local injection and extracorporeal shock wave therapy in post-stroke hemiplegic shoulder pain: a randomized clinical trial	NA	NA	Type II mucosal iodine	NA
2019	Zhang et al.	The Effectiveness of Ultrasound-Guided Steroid Injection Combined with Miniscalpel-Needle Release in the Treatment of Carpal Tunnel Syndrome vs. Steroid Injection Alone: A Randomized Controlled Study	NA	NA	NS	NA

[a] at least two minutes to ensure complete sterilization

[b] non-sterile us gel inside the sterile glove, sterile us gel between the probe and the skin

[c] on a sterile drape

[d] five applications
