# Supplementary material for: A Scoping Review of Clinical Studies on Procedures of Ultrasound-Guided Injection to Ensure Hygiene and Safety
Source: Healthcare (Basel). 2025 May 16;13(10):1165. doi: 10.3390/healthcare13101165 (PMC12110851; doi:10.3390/healthcare13101165)
Supplement: Supplementary file 1 [file healthcare-13-01165-s001.zip › Table S1.pdf]

Table S1. Search strategies

Database	Research equations	Number of results
MEDLINE (via PubMed)	(“ultrasonography”[Title/Abstract] OR “ultrasound”[Title/Abstract] OR “ultrasound-guided”[Title/Abstract] OR “sonography”[Title/Abstract]) AND (“injection”[Title/Abstract] OR “pharmacopuncture”[Title/Abstract]) AND (“randomized controlled trial”[Title/Abstract] OR “controlled clinical trial”[Title/Abstract] OR “double blind method”[Title/Abstract] OR “single blind method”[Title/Abstract] OR “clinical exam”[Title/Abstract] OR “clinical trial”[Title/Abstract] OR “clinical research”[Title/Abstract])	655
Cochrane Central Register of Controlled Trials	(“ultrasound-guided”[Title/Abstract] OR “sonography”[Title/Abstract]) AND (“injection”[Title/Abstract] OR “pharmacopuncture”[Title/Abstract]) AND (“clinical exam”[Title/Abstract] OR “clinical trial”[Title/Abstract] OR “clinical research”[Title/Abstract]) AND (“systematic review”[Title/Abstract] OR “meta-analysis”[Title/Abstract])	766
ScienceON	(“초음파” AND “주사”) OR (“초음파” AND “약침”)	244
Koreanstudies Information Service System	(“초음파” AND “주사”) OR (“초음파” AND “약침”)	53
