# Supplementary material for: A Scoping Review of Clinical Studies on Procedures of Ultrasound-Guided Injection to Ensure Hygiene and Safety
Source: Healthcare (Basel). 2025 May 16;13(10):1165. doi: 10.3390/healthcare13101165 (PMC12110851; doi:10.3390/healthcare13101165)
Supplement: Supplementary file 1 [file healthcare-13-01165-s001.zip › Table S2.pdf]

Table S2. The diseases of participants classified by the ICD-11

Diseases	Frequency
**15. Diseases of the musculoskeletal system or connective tissue**	**59**
Rotator cuff disease (tendinopathy, tear, pain)	9
Osteoarthritis (hip, knee)	8
Subacromial subdeltoid bursitis	7
Plantar Fasciitis	6
Adhesive Capsulitis	5
Facet Joint Syndrome (neck, lumbar)	4
Lateral epicondylitis	3
Acromioclavicular arthropathy	3
Achilles or patellar tendinopathies	2
Impingement Syndrome	2
Pes anserinus tendinobursitis	1
Hallux Rigidus	1
de Quervain's disease	1
trigger finger disease	1
Gluteus medius/minimus tendinopathy	1
Rheumatoid arthritis (ankle, foot joints)	1
Juvenile idiopathic arthritis (knee)	1
Chronic Lateral Ankle Instability (after surgery)	1
Achilles enthesitis (ankylosing spondylitis)	1
Spondyloarthropathy	1
**08. Diseases of the nervous system**	**16**
Carpal Tunnel Syndrome	10
Low back pain with radiculopathy	4
Morton neuroma	1
Piriformis Syndrome	1
**21. Symptoms, signs or clinical findings, not elsewhere classified**	**7**
Myofascial Pain Syndrome (upper trapezius, neck, back)	3
Hemiplegic Shoulder Pain	2
Chronic Coccydynia	1
Adductor-related groin pain	1
**22. Injury, poisoning or certain other consequences of external causes**	**2**
Ribs Fracture	2
**02. Neoplasms**	**1**
Bladder cancers (undergoing transurethral resection of bladder tumor)	1
**Other**	**1**
NA (Comparison of two approaches for shoulder joint injections)	1
